# Extent and characteristic of relationships in canal dimension and canal body ratio between cervical and lumbar spine

**DOI:** 10.1038/s41598-021-98038-0

**Published:** 2021-09-16

**Authors:** Jung-Hee Lee, Kyung-Chung Kang, Ki-Tack Kim, Yong-Chan Kim, Tae-Soo Chang

**Affiliations:** 1 Department of Orthopaedic Surgery, Kyung Hee University Hospital, College of Medicine, Kyung Hee University, 23 Kyungheedaero, Dongdaemun-gu, Seoul, 02447 Republic of Korea; 2 Department of Orthopaedic Surgery, Kyung Hee University Hospital at Gangdong, College of Medicine, Kyung Hee University, Seoul, Republic of Korea

**Keywords:** Bone, Orthopaedics

## Abstract

A known prevalence of concurrent cervical and lumbar spinal stenosis was shown to be 5–25%, but there is a lack of evidence regarding direct relationships in canal dimension and canal-body ratio between cervical and lumbar spine. Total 247 patients (mean age: 61 years, male: 135) with cervical and lumbar computed tomography scans were retrospectively reviewed. Midsagittal vertebral body and canal diameters in reconstructed images were measured at all cervical and lumbar vertebrae, and canal-body ratios were calculated. The canal diameter and ratio were also compared according to the gender and age, and correlation analysis was performed for each value. There were significant correlations between cervical (C3–C7) and lumbar (L1–L5) canal dimension (*p* < 0.001). C5 canal diameter was most significantly correlated with L4 canal diameter (r = 0.435, *p* < 0.001). Cervical canal-body ratios (C3–C7) were also correlated with those of lumbar spine (L1–L5) (*p* < 0.001). The canal-body ratio of C3 was most highly correlated with L3 (r = 0.477, *p* < 0.001). Meanwhile, mean canal-body ratios of C3 and L3 were significantly smaller in male patients than female (*p* = 0.038 and *p* < 0.001) and patient’s age was inversely correlated with C5 canal diameter (r = − 0.223, *p* < 0.001) and C3 canal-body ratio (r = − 0.224, *p* < 0.001). Spinal canal dimension and canal-body ratio have moderate degrees of correlations between cervical and lumbar spine and the elderly male patients show the tendency of small canal diameter and canal-body ratio. This relationship of cervical and lumbar spine can be an important evidence to explain to the patients.

Tandem spinal stenosis (TSS) is caused by the simultaneous involvement of the cervical and lumbar spines. Since Dagi et al. first described TSS, there has been growing interest in the relationship between the cervical and lumbar spine and proper evaluation and treatment of the TSS^1–3^. The TSS is known to occur in 5–25% in previous literature^4,5^. Because the patient with tandem spinal stenosis shows cervical and lumbar stenotic symptoms simultaneously, physicians should distinguish between the symptoms of cervical spinal problems and lumbar spinal problems and apply the proper remedy^6–8^.

Bajwa et al.^4^ and Lee et al.^9^ proved that congenital stenosis of the cervical spine was associated with congenital stenosis of the lumbar spine, using adult skeletal specimens. However, although they reported that about 15–30 percentage of patients showed combined cervical and lumbar spinal stenosis, they did not show extent of the relationships in canal dimension and canal-body ratio between cervical and lumbar spine and special features regarding their relationships. Congenital spinal stenosis can be considered as if it were of less clinical importance, because spinal canal stenosis usually occurs in the intervertebral disc level. However, congenital canal stenosis is a known risk factor for acute spinal cord injury and age-related development of degenerative spinal disease^10^ and the bony spinal canal is known to be narrower with age^11,12^. Therefore, the narrow bony spinal canal is thought to be no less important than degenerative change at the intervertebral disc level. and various studies on congenital stenosis itself are necessary.Until now, previous studies mainly focused on the prevalence and appropriate management of the TSS symptoms and signs^2,13–15^, but there are few reports for assessing the extent of cervical and lumbar spinal inter-relationship. The aim of this study is to verify direct relationships in canal dimension and canal-body ratio between cervical and lumbar spine and to evaluate the characteristics according to the patients’ age and gender, using cervical and lumbar computed tomography (CT) scans.

## Methods

### Patient populations

A total of 284 patients that visited our institution between Jan. 2013 and Apr. 2017 and had simultaneous cervical and lumbar CT scans regardless of their diagnosis were retrospectively reviewed. Younger patients (< 20 years old) and patients with definite ossification of posterior longitudinal ligament, spondylolysis, deformity of vertebral body, spinal fracture, or inflammatory disease such as ankylosing spondylitis, were excluded from this study. Also, patients with films done outside our institution were not included. Finally, 247 patients (mean age: 61 (21–82), male: 135) were included in this study.

For evaluation of canal stenosis, vertebral body and canal diameters were measured at the cervical (C3-7) and the lumbar (L1-5) vertebrae, and spinal canal to vertebral body ratios were calculated at all levels. The canal diameter and ratio were also analyzed according to the patient’s age and gender, and correlation analysis was performed for each value.

### Radiographic measurements

**Figure 1:**
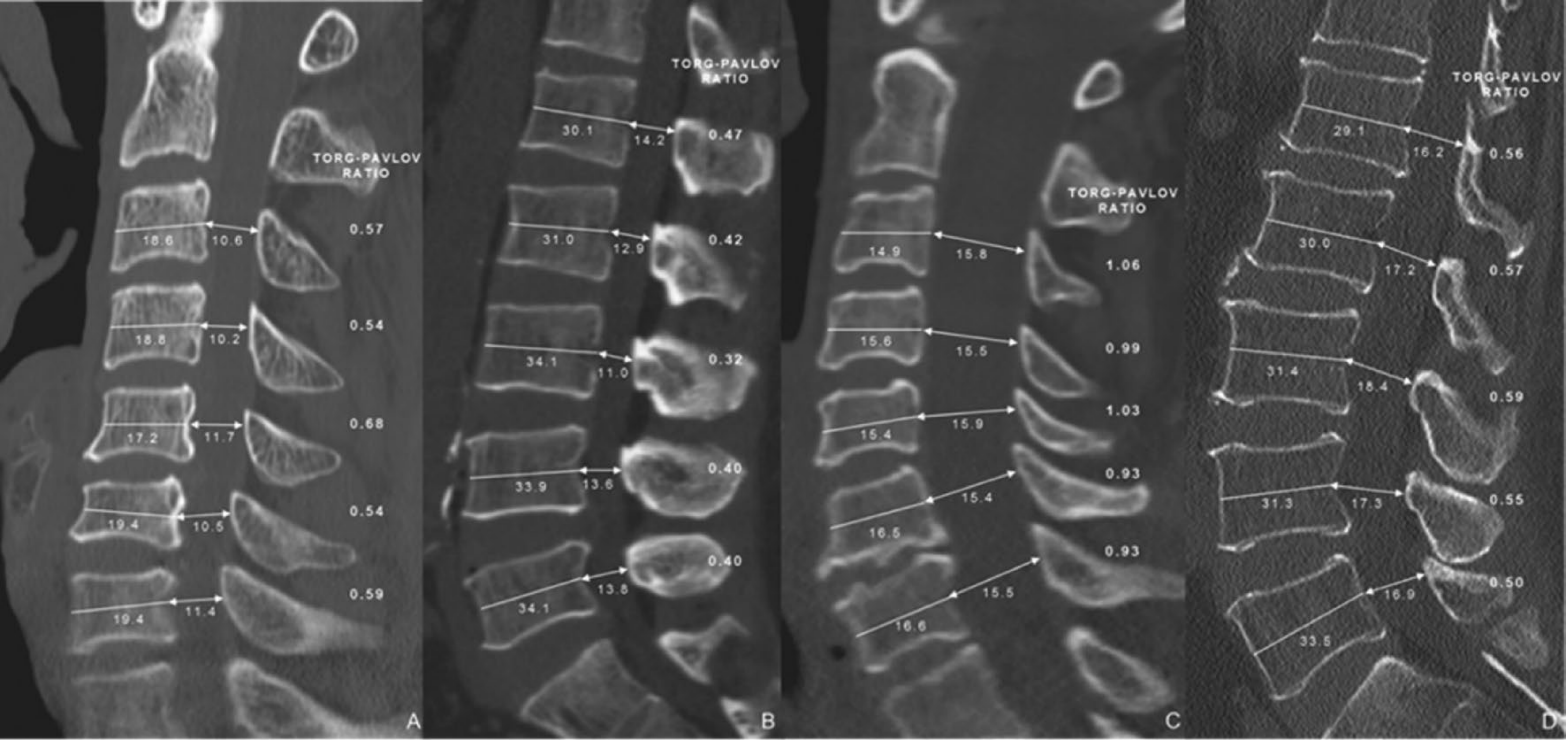
Representative images of two cases. (**A**,**B**) A 51-year-old male patient shows concurrent cervical and lumbar spinal canal stenosis. Mean spinal canal to vertebral body ratios were 0.58 (cervical) and 0.40 (lumbar). (**C**,**D**) Another 59 year-old female patient represents large canal diameter and high canal to body ratio (0.99: cervical and 0.55: lumbar).

In this study, CT scans performed using Ingenuity CT (2.5 mm thickness, Philips Medical System Inc, 595 Miner road, Cleveland, Ohio 44143, USA) and reformatted images of CT scans were used instead of conventional lateral radiographs. The following measurements were taken for the C3–C7 and L1–5 levels on sagittal views of the reformatted images of CT scans: the midsagittal diameter of the vertebral body and the canal diameter. Midsagittal slice that had the widest vertebral body and canal around a square center line was selected and the diameter of the vertebral body between the midpoints of anterior surface and the midpoint of posterior surface and the canal diameter between the midpoint of the posterior surface of the vertebral body and the nearest part of opposite lamina were measured in double magnified sagittal images (Fig. 1)^16^.

To minimize inter- and intra-observer errors, two independent orthopedic surgeons evaluated the digital radiographs, which were uniformly magnified twice. Inter- and intra-observer intra-class correlation coefficients (ICCs) were assessed for the vertebral body and canal diameters.

### Statistical analysis

Statistical analysis was performed by a professional medical statistical consultant using SPSS version 19.0 statistical software (IBM Corp, Armonk, New York). Values were recorded as mean ± standard deviation. Depending on the normality of the data, correlations among the measured variables were analyzed by Pearson’s product-moment or Spearman’s rank correlation coefficient. An independent-samples *t-*test or Mann- Whitney *U-*test was used to compare parameters. Significance was accepted for a *p* value of less than 0.05.

### Ethical consideration and approval

This study has been approved by the institutional review boards at Kyung Hee University hospital and patient’s informed consent is waved (KHUH 2017-05-077-003). All patients’ data were made anonymous and kept confidential. All procedures were indicated and performed in compliance with our department’s standards and the Declaration of Helsinki.

## Results

Inter- and intra-observer reproducibilities were high for vertebral body and canal diameter measurement. Inter-observer ICCs for vertebral body and canal diameter measurement were 0.821 and 0.879, respectively, and the corresponding intra-observer ICCs were 0.912 and 0.920, respectively.

The mean measurements of the sagittal diameter of the vertebral body (C3-C7) were 16.6 ± 1.8 mm (C3), 16.5 ± 2.0 mm (C4), 16.5 ± 2.4 mm (C5), 17.4 ± 2.3 mm (C6), and 17.4 ± 2.2 mm (C7). The mean measurements of cervical canal diameter (C3-C7) were 12.1 ± 1.5 mm (C3), 11.9 ± 1.7 mm (C4), 12.1 ± 1.9 mm (C5), 11.9 ± 1.9 mm (C6), and 12.2 ± 1.9 mm (C7). The cervical 6^th^ and 7^th^ vertebral body diameters are significantly larger than the dimeters of C3-C5 vertebral body (*p* < 0.001), but cervical canal diameters are similar in all cervical segments (C3-C7). The mean measurements of sagittal diameter of the vertebral body (L1-L5) were 30.0 ± 2.9 mm (L1), 31.6 ± 3.0 mm (L2), 33.1 ± 3.0 mm (L3), 33.4 ± 2.9 mm (L4), and 33.1 ± 3.1 mm (L5). The mean measurements of lumbar canal diameters (L1-L5) were 14.1 ± 1.7 mm (L1), 13.8 ± 1.6 mm (L2), 13.4 ± 1.8 mm (L3), 14.1 ± 1.9 mm (L4), and 14.6 ± 2.4 mm (L5). Similar to the cervical vertebrae, lumbar vertebral body diameters are significantly larger in L3-L5 than those of L1-L2 (*p* < 0.001), but lumbar canal diameters are similar in L1-L5 vertebrae.

The cervical canal diameters (C3-C7) were significantly correlated with lumbar canal diameters at all segments (L1-L5) (*p* < 0.001). The C5 cervical canal diameter showed the highest significant correlation with the L4 canal diameter (r = 0.435, *p* < 0.001) (Table 1).

**Table 1:** Correlations between cervical canal and lumbar canal diameters.

Correlation coefficient(r)*	L1 *(14.07 ± 1.70)	L2 (13.76 ± 1.64)	L3 (13.39 ± 1.79)	L4 (14.07 ± 1.93)	L5 (14.61 ± 2.37)
C3 (12.07 ± 1.49)	0.340	0.352	0.370	0.394	0.352
C4 (11.90 ± 1.69)	0.313	0.334	0.371	0.392	0.346
C5 (12.07 ± 1.87)	0.327	0.385	0.425	0.435^§^	0.351
C6 (11.88 ± 1.92)	0.351	0.342	0.379	0.377	0.298
C7 (12.16 ± 1.91)	0.347	0.331	0.372	0.313	0.285

All cervical canal diameters from C3 to C7 were correlated with lumbar canal diameters at all segments. Particularly, the C5 canal diameter showed highest correlation with L4 canal diameter.

All *p* value < 0.001, * unit : mm, ^§^highest correlation coefficient.

Meanwhile, the mean canal-body ratios of the cervical spine (C3-C7) were 0.74 ± 0.14 (C3), 0.73 ± 0.16 (C4), 0.75 ± 0.17 (C5), 0.70 ± 0.17 (C6), and 0.71 ± 0.15 (C7). The mean lumbar canal-body ratios (L1-L5) were 0.47 ± 0.08 (L1), 0.44 ± 0.08 (L2), 0.41 ± 0.08 (L3), 0.43 ± 0.07 (L4), and 0.45 ± 0.09 (L5). The cervical canal-body ratios of cervical spine (C3-C7) were also significantly correlated with those of lumbar spine at all segments (L1-L5) (*p* < 0.001). The canal-body ratios of the C3 (0.74 ± 0.14) were most significantly correlated with those of the L3 (0.41 ± 0.08) (r = 0.477, *p* < 0.001) (Table 2).

**Table 2:** Correlations between cervical and lumbar spinal canal-body ratios.

Correlation coefficient(r)*	L1 (0.47 ± 0.08)	L2 (0.44 ± 0.08)	L3 (0.41 ± 0.08)	L4 (0.43 ± 0.07)	L5 (0.45 ± 0.09)
C3 (0.74 ± 0.14)	0.415	0.430	0.477^§^	0.462	0.386
C4 (0.73 ± 0.16)	0.363	0.405	0.455	0.441	0.356
C5 (0.75 ± 0.17)	0.338	0.403	0.437	0.427	0.331
C6 (0.70 ± 0.17)	0.351	0.367	0.417	0.381	0.275
C7 (0.71 ± 0.15)	0.364	0.365	0.413	0.352	0.333

All cervical canal-body ratios from C3 to C7 were correlated with lumbar canal-body ratios at all segments. Particularly, the C3 canal-body ratio showed highest correlation with L3 canal-body ratio.

*All *p* value < 0.001, ^§^highest correlation coefficient.

**Figure 2:**
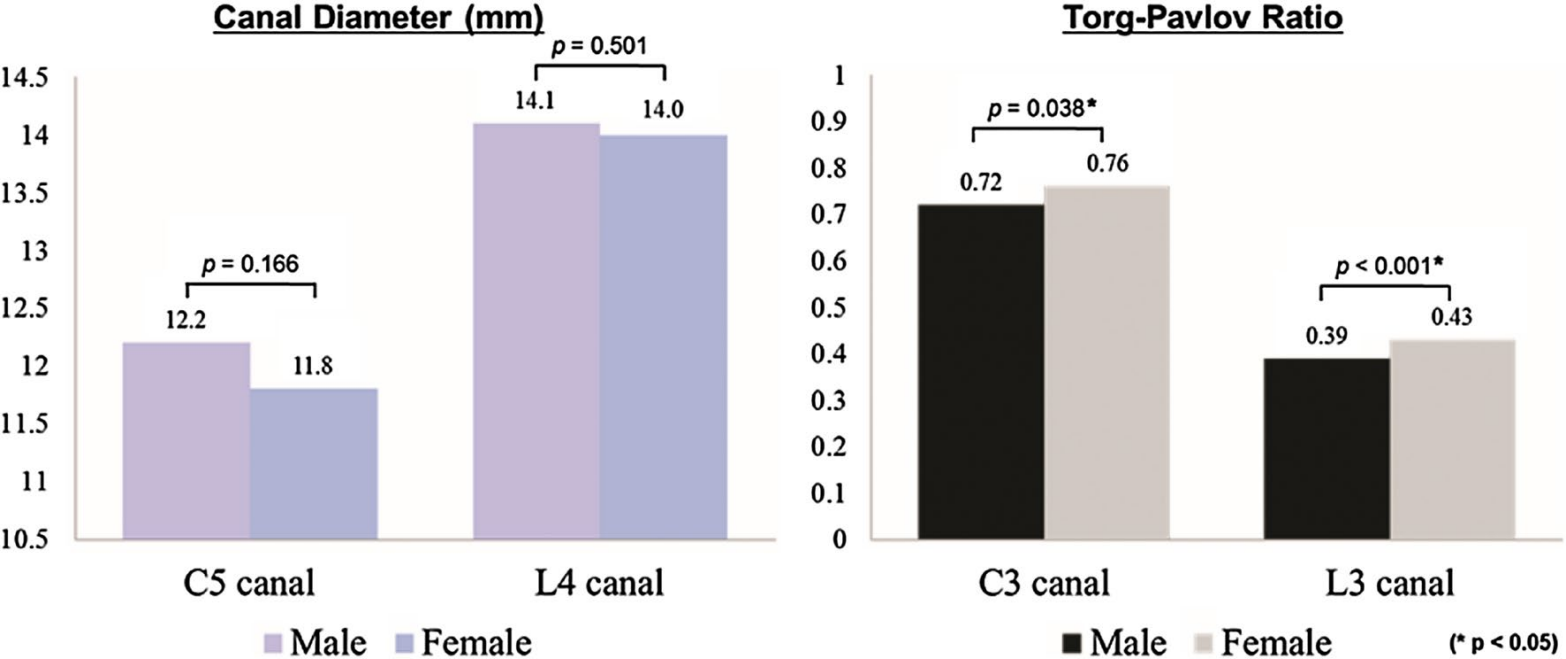
Comparisons of canal diameter and spinal canal to body ratio between male and female patients. There were significant differences in the canal to body ratios between male and female patients (*p* < 0.05, right graph), but not in canal diameters (*p* > 0.05, left graph).

In comparisons between male and female patients, the C5 and L4 canal diameters were not significantly different (*p* > 0.05), but male patients showed significantly lower mean canal-body ratios than female patients (C3:0.72 vs. 0.76, *p* = 0.038 and L3: 0.39 vs. 0.44, *p* < 0.001) (Fig. 2). Uniquely, the patient’s age was inversely correlated with the C5 canal diameter (r = − 0.201, *p* = 0.002) and the C3 canal-body ratio (r = − 0.243, *p* < 0.001), but did not show a significant relationship with the L4 canal diameter or the canal-body ratio of the L3 (Table 3). The spinal canal stenosis is more remarkable in male and elderly patients than in female and young patients.

**Table 3:** Correlations between age and mean canal diameter/canal-body (Torg-Pavlov) ratio.

Correlation coefficient (r)	Canal Diameter		Torg-Pavlov Ratio	
	C5	L4	C3	L3
Age	− 0.201*	− 0.115	− 0.243*	− 0.066
*p* value	0.002	0.084	0.000	0.312

The patients age was inversely correlated with C5 canal diameter and C3 canal-body ratio.

*p value < 0.05.

## Discussion

### Key results and Interpretation

In previous studies, mainly the prevalence of tandem stenosis and its treatment were discussed^17^. Lee et al.^9^ described the prevalence of tandem stenosis and the associative relationship between lumbar and cervical stenosis through a cadaveric study. In that study, 440 cadavers were used, and this large cadaveric population is an important advantage. A total of 21.5% (95/440) of cervical stenosis, 16.8% (74/440) of lumbar stenosis and 5.4% (24/440) of tandem stenosis were identified. However, in this study, detailed information about direct relationships between cervical and lumbar canal diameters and ratios was not presented^9,18^ and there were no clinical considerations including patients’ age, gender, degree of degeneration and so on.

In our study, the degree of correlations between actual cervical and lumbar canal stenosis was evaluated. The canal diameters and spinal canal to vertebral body ratios between cervical and lumbar spinal canal showed moderate degrees of correlations. The highest correlation coefficient was 0.435 between the C5 and L4 canal diameter (*p* < 0.001) and 0.477 between the C3 and L3 canal to body ratio (*p* < 0.001), respectively. Particularly, our results show the most relevant segment of cervical and lumbar spine and provide beneficial information to help choose the representative segment to predict the extent of tandem spinal stenosis. Meanwhile, patient’s age had a negative correlation with the canal diameter and canal to body ratio similar to other studies^11,12^, and mean canal to body ratio was significantly lower in the male patients than female patients. The cervical and lumbar canal diameter and canal-body ratio have significant correlations and particularly the elderly male patients are more likely to have a higher risk of congenital canal stenosis. These results would be important evidences for predicting future spinal diseases in patients with congenital canal stenosis.

From a clinical perspective, symptomatic spinal canal stenosis usually occurs in the disc level, not in the mid-body. However, congenital canal stenosis at the mid-vertebral body level also has important roles associated with patients’ critical situations, such as acute spinal cord injury or age-related development of degenerative cervical myelopathy^10,19,20^. Nouri et al. also reported that the patients with congenital spinal stenosis developed myelopathy at a younger age and had greater impairment and disability due to a lower threshold of degenerative changes for spinal cord compression and less cerebrospinal fluid surrounding the spinal cord^19^. Furthermore, Morishita et al. showed the relationship between the cervical spinal canal diameter (less than 13 mm) and pathological changes in the cervical spine. This result means that kinematics with congenitally narrow canal may greatly contribute to pathological changes^21^. In this sense, the degree of congenital spinal stenosis and canal-body ratio at the mid-body level (shortening of pedicles) is thought to have a critical importance in anticipating future development of spinal diseases.

### Points of difference

We used the spinal canal to vertebral body ratio^22,23^ to identify a correlation between cervical and lumbar canal diameters and the degree of its relationship. The value is calculated by dividing the mid-vertebral sagittal diameter of the cervical spinal canal by the sagittal diameter of the vertebral body. Conventionally, the Torg-Pavlov ratio is measured using conventional lateral radiographs in cervical spine, but a lumbar spinal canal diameter cannot be measured exactly, due to the overlapping shadows of the pedicle and lamina and osteophyte formation, and ossification around the vertebral body may impede the accurate measurement of the canal diameter. The authors thought that the reformatted sagittal images of CT scans could reduce errors and provide more accurate measurements than x-rays or magnetic resonance images.

Interestingly, there were significant differences in spinal canal diameter and Torg-Pavlov ratio according to the patients’ ages and genders. A patient’s age was inversely correlated with the cervical canal diameter and canal to body ratio, but not with the lumbar canal or ratio. Meanwhile, male patients showed significantly lower canal to body ratios than female patients. With these results, although the exact causes could not be explained, the authors carefully concluded that elderly male patients were vulnerable to cervical canal stenosis. To clarify the cause of these results, a large-scale epidemiological study is necessary.

Although there are studies on the importance of the cervical-pelvic relationship, few papers have found its definite association^24^. In this study, we have found that the size of the cervical and lumbar canal and canal-body ratio are inherently related. This result is considered to be important evidence not only for tandem spinal stenosis between the cervical and lumbar spines but for explaining similar characteristics of the cervical and lumbar spine, even if there are no additional images of other spinal department. Embryologically, the cervical and lumbar spines begin similarly, as secondary curves are developed. Therefore, cervical and lumbar spinal association is a matter of course. Until now, there has been a lack of evidence for the direct relationship between cervical and lumbar spinal features. In this sense, the authors’ result would be an important evidence for explaining the interrelationship between the cervical and lumbar spinal problems.

### Study limitations

There were some weaknesses in this study. Firstly, this study evaluates only radiographic results, not including the data related to clinical symptoms. However, there were some studies for symptomatic tandem spinal stenosis and in this study, we have focused spinal canal and body diameter without significant degeneration and its ratio with using the reformatted images of CT scans. Although the CT scan don’t reveal disc degeneration or ligament hypertrophy associated with clinical symptoms, it can present congenital canal stenosis itself without degenerative changes and accurately measure spinal canal and vertebral body, compared to using the x-ray or MRI^25^. Secondly, all the cases of this study have visited our hospital regardless of the patients’ diagnosis and had both cervical and lumbar CT scans. There can be a bias in patients’ selection. However, unlike previous studies, the purpose of this study is not to show a prevalence of tandem spinal stenosis compared to normality data, but to analyze degree of the relationships in canal dimension and canal-body ration between cervical and lumbar spine and to verify its differences according to age and gender. Therefore, the authors thought that this error would not have significant influence on the results.

## Conclusion

In conclusion, the authors confirmed that cervical canal diameter and canal-body ratio are significantly associated with lumbar canal diameter and ratio. Particularly, male patients showed lower canal-body ratios in the cervical and lumbar spines than female patients, and patient age was negatively correlated with canal diameter and canal-body ratio in the cervical spine. A prospective study with larger and standardly selected patients will be warranted.
